# MiR-485 targets the *DTX4* gene to regulate milk fat synthesis in bovine mammary epithelial cells

**DOI:** 10.1038/s41598-021-87139-5

**Published:** 2021-04-07

**Authors:** Juan Liu, Ping Jiang, Ambreen Iqbal, Shaokat Ali, Zhen Gao, Ziyi Pan, Lixin Xia, Fuquan Yin, Zhihui Zhao

**Affiliations:** 1 College of Coastal Agricultural Sciences, Guangdong Ocean University, Zhanjiang, 524088 Guangdong People’s Republic of China; 2 College of Animal Science, Jilin University, Xi An Road 5333, Changchun, 130062 Jilin People’s Republic of China

**Keywords:** Cell biology, Molecular biology

## Abstract

MicroRNAs (miRNAs) are mRNA suppressors that regulate a variety of cellular and physiological processes, including cell proliferation, apoptosis, triglyceride synthesis, fat formation, and lipolysis, by post-transcriptional processing. In previous studies, we isolated and sequenced miRNAs from mammary epithelial cells from Chinese Holstein cows with high and low milk fat percentages. MiR-485 was one of the significantly differentially expressed miRNAs that were identified. In the present study, the relationship between the candidate target gene *DTX4* and miR-485 was validated by bioinformatics and real-time fluorescent quantitative PCR (qRT-PCR) and Western blot (WB) analyses in bovine mammary epithelial cells (bMECs). The results indicated that miR-485 negatively regulated the mRNA expression of the target gene *DTX4*. Furthermore, an shRNA interference vector for the target gene *DTX4* was constructed successfully, and it increased the triglyceride content and reduced the cholesterol content of transfected cells. These results suggest that miR-485 may affect the contents of triglycerides (TGs) and cholesterol (CHOL) by targeting the *DTX4* gene. This study indicates that miR-485 has a role in regulating milk fat synthesis and that miR-485 targets the *DTX4* gene to regulate lipid metabolism in bMECs. These findings contribute to the understanding of the functional significance of miR-485 in milk fat synthesis.

## Introduction

Milk is secreted from the mammary glands of mammals and is a white-colored nutrient-rich liquid produced as the primary source of nutrition for infant mammals^1^. In 2011, dairy farms worldwide had approximately 730 million tons of milk from 260 million dairy cows (FAO, May 2012). According to the estimation of the FAO, in 2011, 85% of all the milk produced worldwide came from cows^2^. With the change in consumer demand, the likelihood of consuming milk fat, considered a good source of fat, has increased, and milk fat is used as an indicator of milk quality. The major constituents of milk fat are triglycerides (TGs), which comprise approximately 97–98% milk fat content. The other contents of milk fat include fatty acids (FAs) and cholesterol (CHOL) as minor components^3,4^. TGs are an important part of milk, and their synthesis in bovine mammary epithelial cells (bMECs) affects milk fat percentage^3,5^.

One of the molecular mechanisms regulating milk fat production involves the role of miRNAs in bMECs. MiRNAs are a class of non-coding RNAs that are approximately 22 nucleotides long, are fully or partially complementary to target mRNAs, and promote the degradation of target genes or the inhibition of translation, thereby enabling the regulation of target gene expression levels^6^. The expression of miRNAs has a developmental stage and tissue specificity; miRNAs are also expressed in the mammary gland and play essential roles in regulating mammary gland development, lipid metabolism, and lactation^7^. Studies have indicated that many miRNAs are involved in fat formation and lipolytic action^7^. For example, miR-224 overexpression decreased TGs synthesis and promoted apoptosis. Bta-miR-29b promoted TGs production and suppressed apoptosis^7^. Additionally, miR-33a, miR-21, miR-23a, and miR-877 have been reported to affect TGs production and apoptosis of bMECs^8^.

In a previous study, we performed miRNA sequencing on the bMECs of Chinese Holstein dairy cows with a high or low milk fat percentage^9^ and selected miR-485 from the differentially expressed miRNAs to verify its role in milk fat metabolism. Previously, miR-485-5p was found to modulate mitochondrial fission by targeting mitochondrial-anchored protein ligase in cardiac hypertrophy^10,11^. MiR-485 mediated the response of human mesangial cells (HMCs) to high glucose (HG) concentrations. Overexpression of miR-485 suppressed HG-induced proliferation of HMCs^12,13^. Emerging evidence suggests that miR-485 plays a critical role in inflammation and is involved in the development and progression of inflammatory diseases, including osteoarthritis and cutaneous lupus erythematosus^14^. MiR-485 also plays a role in neurological diseases^15^, and it might play an important role in breast cancer by suppressing cell proliferation and migration^16^. MiR-485-5p decreased cancer progression and enhanced chemosensitivity, thereby regulating survival in breast cancer^17^. Kang et al. found that miR-485 decreased gastric cancer cell proliferation, invasion, and tumorigenesis by downregulating the Flot1 gene^18^.

The miRNAs involved in the regulation of milk fat synthesis were identified in a screen, and the sequencing results showed that miR-485 is a differentially expressed miRNA. Upon bioinformatic analysis, the differentially expressed miR-485 was found to target the *DTX4* gene. Deltex belongs to a protein family comprised of four members, *DTX1*, *DTX2*, *DTX3*, and *DTX4*, which are recognized as cytoplasmic downstream elements of the notch signaling pathway^19^. Previous studies have suggested that the Deltex family of proteins is closely associated with cell differentiation and cell development. For example, *DTX1* is responsible for B cell progression^20^ and neural differentiation. Overexpression of *DTX2* decreased the expression of myogenin^21^. The more recent study on cell surface ligand binding suggested that *DTX4* was ubiquitylated by the E3 ubiquitin-protein ligase (Protein deltex-4)^22^.

However, despite advancements in biotechnology, little is known about the role of *DTX4* in adipogenesis. This study aimed to determine the effects of miR-485 overexpression or inhibition on TGs, CHOL, and NEFA in bMECs to further explore the regulatory mechanism of miR-485 in lipid metabolism bMECs. Our findings also provide evidence that miR-485 targets the *DTX4* gene to regulate lipid metabolism by suppressing the E3 ubiquitin-protein ligase *DTX4* (Protein deltex-4).

## Results

### Expression trends of miR-485

**Figure 1:**
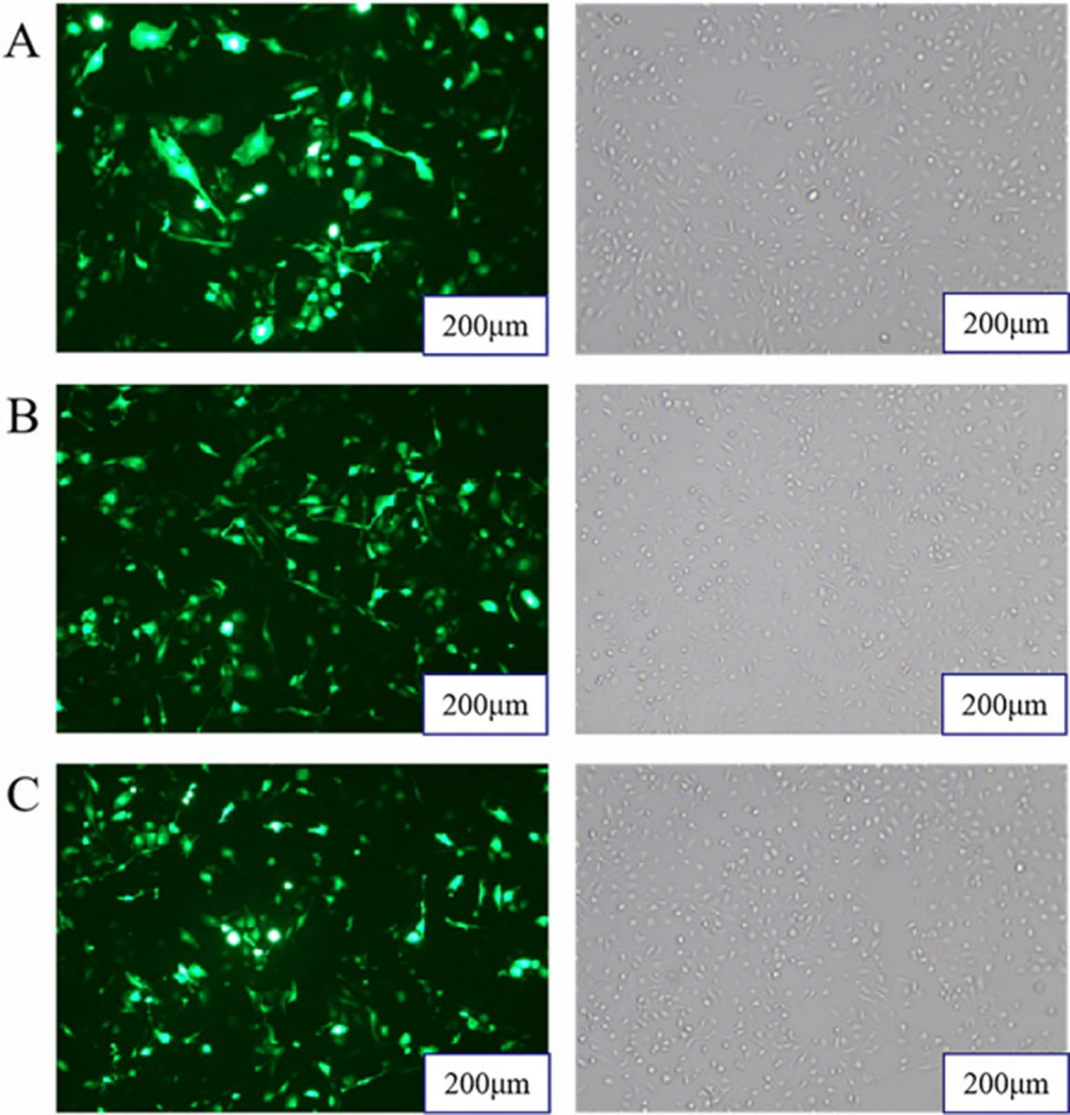
Cells were observed under a fluorescence microscope at 36 h post-transfection. (**A**) miR-485 mimics; (**B**) miR-485 inhibitor; (**C**) miR-485 shNC.

**Figure 2:**
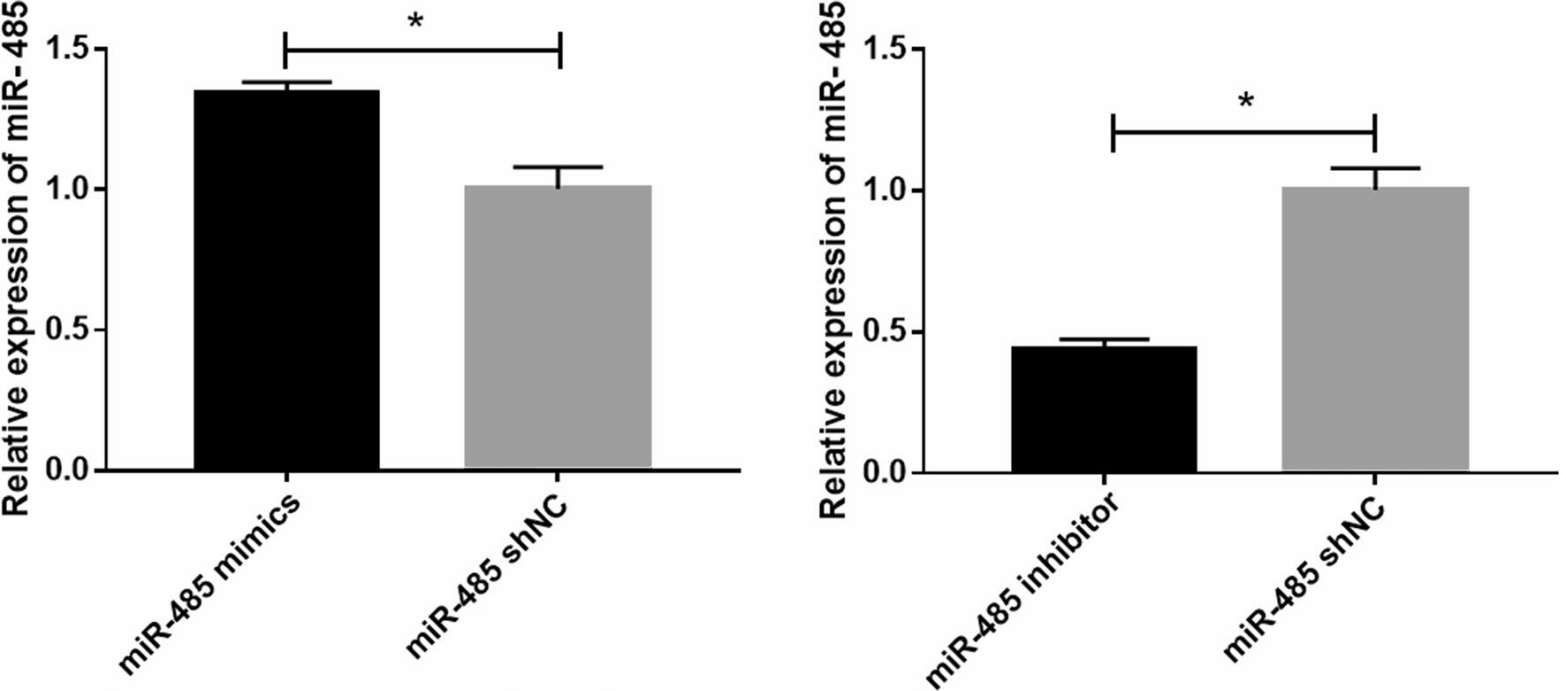
Cells were observed under a fluorescence microscope at 36 h post-transfection. Relative expression of miR-485 (*P < 0.05, **P < 0.01).

Cell morphology and transfection efficiency were observed with a fluorescence microscope. The effect on fluorescence is shown in Fig. 1A–C. miR-485 mimics and inhibitor transfection was successful. After transfection of bMECs with miR-485 mimics, the expression of miR-485 detected by qPCR in the mimics group was significantly higher than that in the shNC group (*P < 0.05). Furthermore, compared with that in the shNC group, the expression of miR-485 in the inhibitor group was significantly reduced (Fig. 2).

### Regulation of TGs, CHOL, and NEFA contents by miR-485 in bMECs

**Figure 3:**
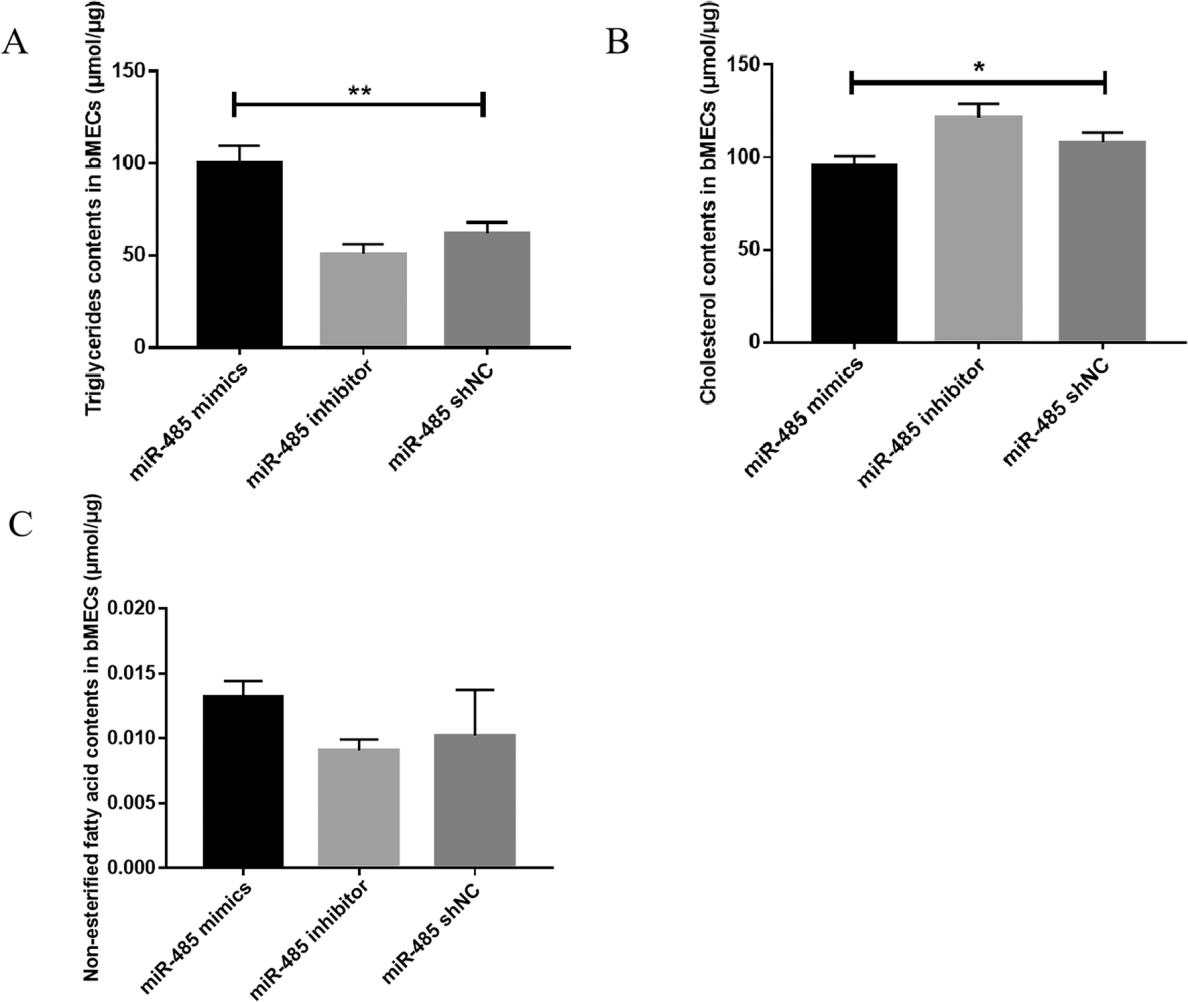
Detection of the TGs, CHOL, and NEFA contents of bMECs transfected with miR-485 mimics, miR-485 inhibitor, or miR-485 shNC (**P < 0.01, *P < 0.05; ns means *P* > 0.05). (**A**) Triglyceride contents in bMECs; (**B**) Cholesterol contents in bMECs; (**C**) Non-esterified fatty acid contents in bMECs.

Compared with that of the bMECs transfected with shNC, the TGs content of the bMECs transfected with miR-485 mimics was significantly upregulated. On the other hand, compared with that in the shNC group, the TGs content in the miR-485 inhibitor group was significantly downregulated. The above data suggest that miR-485 regulated TGs contents in bMECs (Fig. 3A). Additionally, miR-485 was found to regulate CHOL synthesis. Compared with that in the miR-485 shNC group, the CHOL content in the miR-485 mimics group was significantly downregulated (*P < 0.05). Compared with that in the miR-485 shNC group, the CHOL content in the miR-485 inhibitor group was significantly upregulated (Fig. 3B). Compared with that in the miR-485 shNC group, the NEFA content in the miR-485 mimics group was increased, and the NEFA content in the miR-485 inhibitor group was downregulated compared with that in the miR-485 shNC group (*P* > 0.05). (Fig. 3C).

### Relative expression of *DTX4*

**Figure 4:**
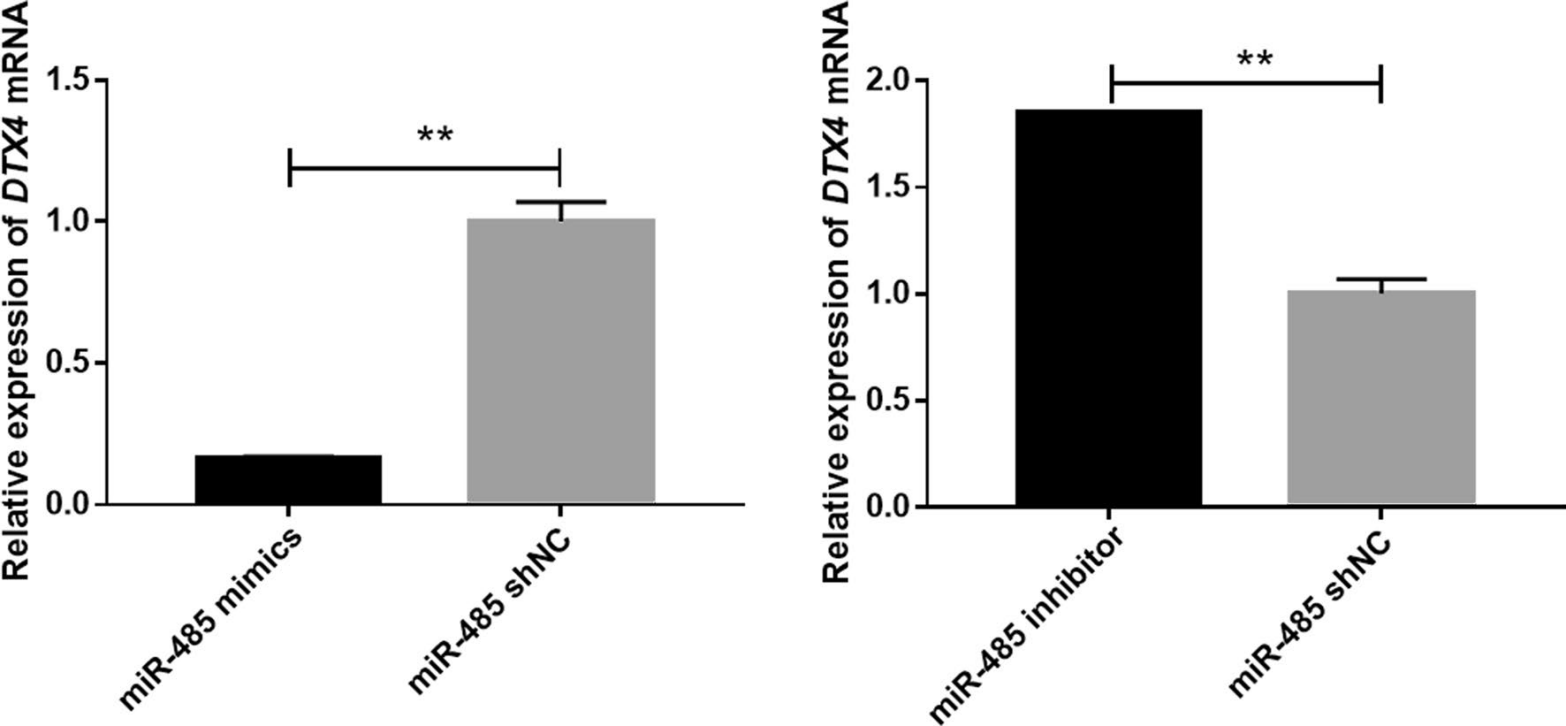
Relative expression of the *DTX4* gene in bMECs after transfection of miR-485 mimics, miR-485 inhibitor, or miR-485 shNC (**P < 0.01).

The relative mRNA expression of *DTX4*, a candidate target gene of miR-485, was significantly lower (**P < 0.01) in bMECs transfected with miR-485 mimics than in bMECs transfected with shNC (Fig. 4). On the other hand, the relative mRNA expression of *DTX4* in bMECs transfected with miR-485 inhibitor was significantly higher (**P < 0.01) than that in bMECs transfected with shNC (Fig. 4). This indicated that miR-485 negatively regulated the mRNA expression level of *DTX4*.

### Analysis of dual-luciferase activity

**Figure 5:**
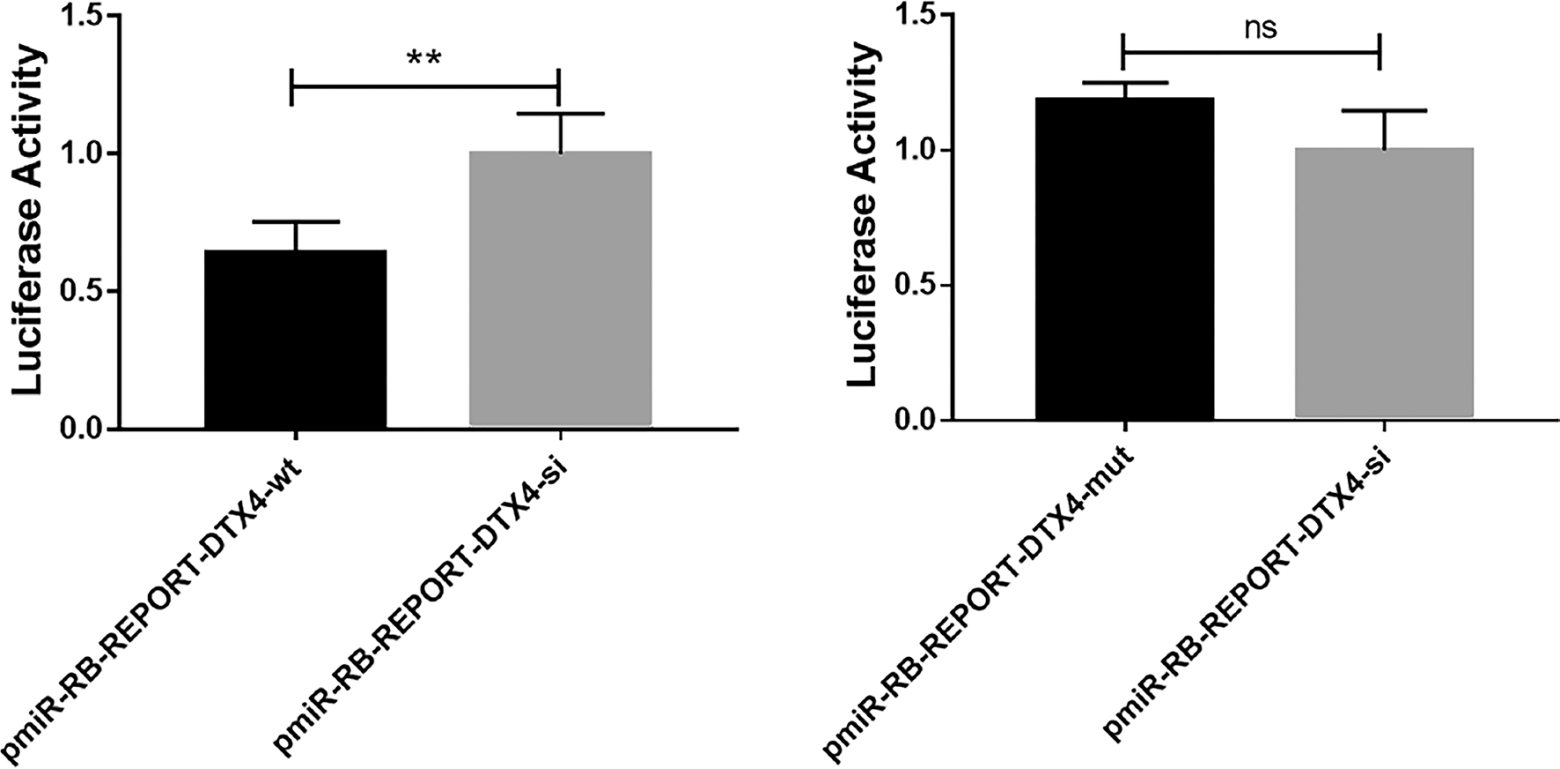
Dual-luciferase activity analysis showing the relative expression of miR-485 and genes after the co-transfection of miR-485 mimics and *DTX4*-wt, miR-485 mimics and *DTX4*-mut, and miR-485 mimics and *DTX4*-si in bMECs (*P < 0.05, **P < 0.01; ns means *P* > 0.05).

More than a dozen target genes related to lipid metabolism were identified by bioinformatics prediction. The TargetScan website predicted that the 3′UTR of the *DTX4* mRNA contained binding sites for miR-485. Verified the miR-485 binding sites in the 3′UTR of *DTX4* mRNA. The results of co-transfection showed that the luciferase activity of bMECs transfected with the *DTX4*-wt vector was significantly lower than that of bMECs transfected with the *DTX4*-si/mut vector, as shown in Fig. 5. These results confirmed that *DTX4* was a target gene of miR-485.

### Western blot analysis

**Figure 6:**
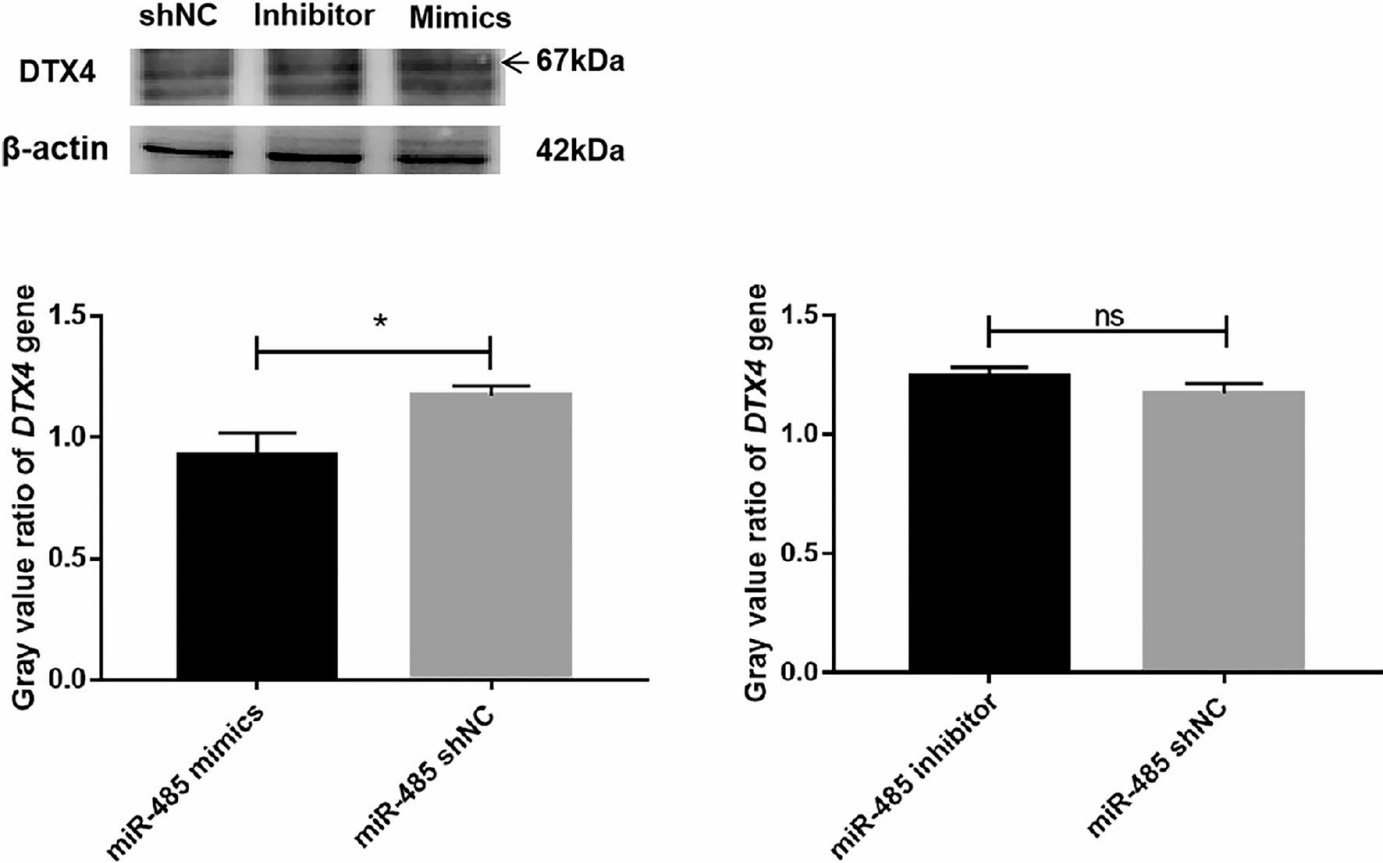
Western blot analysis of the protein expression of the *DTX4* gene in bMECs transfected with miR-485 mimics, miR-485 inhibitors, or miR-shNC (*P < 0.05).

After analyzing the expression of *DTX4* at the mRNA level, we also investigated the protein level of *DTX4* to confirm the regulatory effect of miR-485 on the expression of the *DTX4* gene (Fig. 6). The results suggested that the expression of *DTX4* in the miR-485 inhibitor group was significantly increased compared to that of the miR-485 shNC group. The miR-485 mimics, and miR-485 shNC groups expression was lower than that of the miR-485 inhibitor group. This indicated that miR-485 mimics significantly decreased the expression of *DTX4* at both the mRNA and protein levels. MiR-485 downregulated the expression of the *DTX4* gene.

### Expression of the *DTX4* gene in bMECs after interference

**Figure 7:**
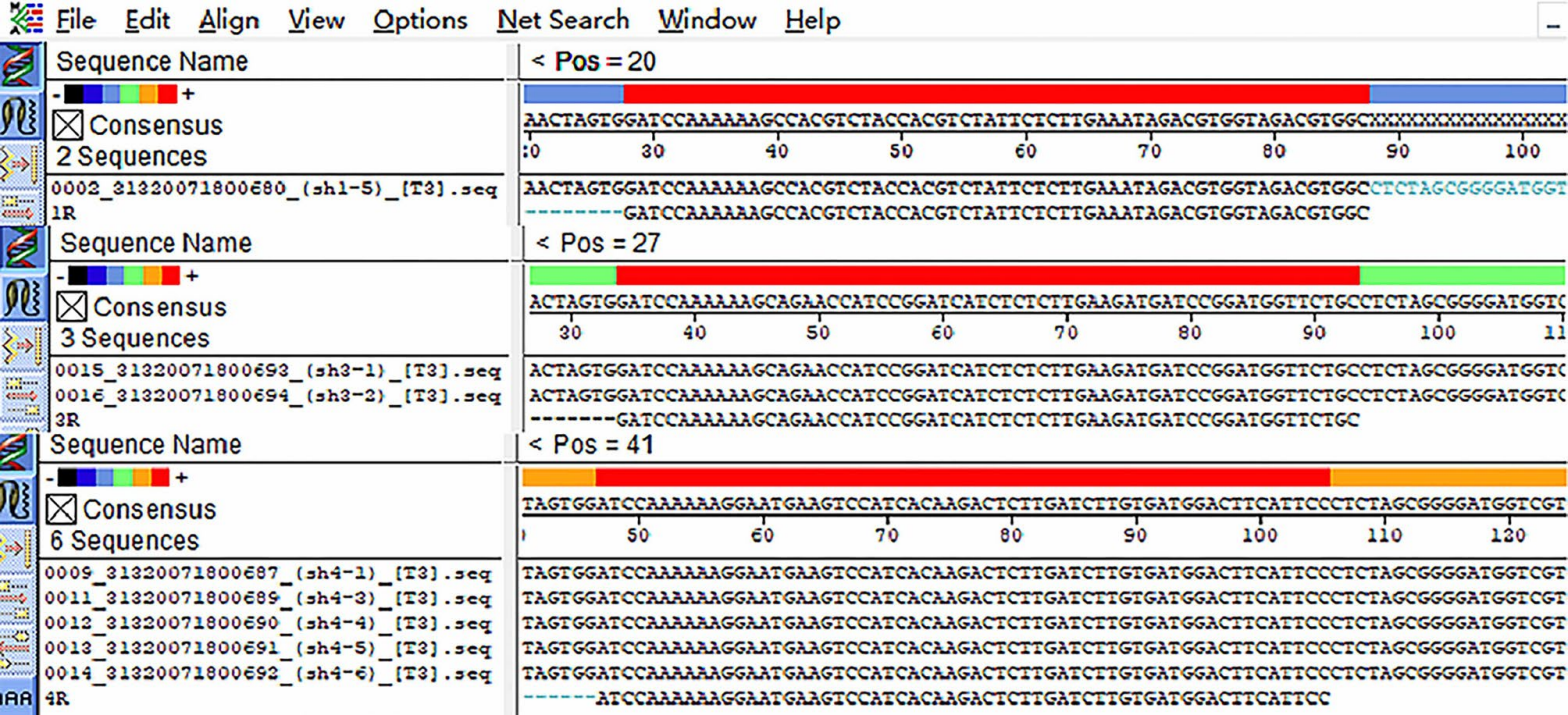
The pb7SK-*DTX4*-shRNA interference vector was successfully constructed by annealing the *DTX4* interference sequence into the pb7SK vector.

**Figure 8:**
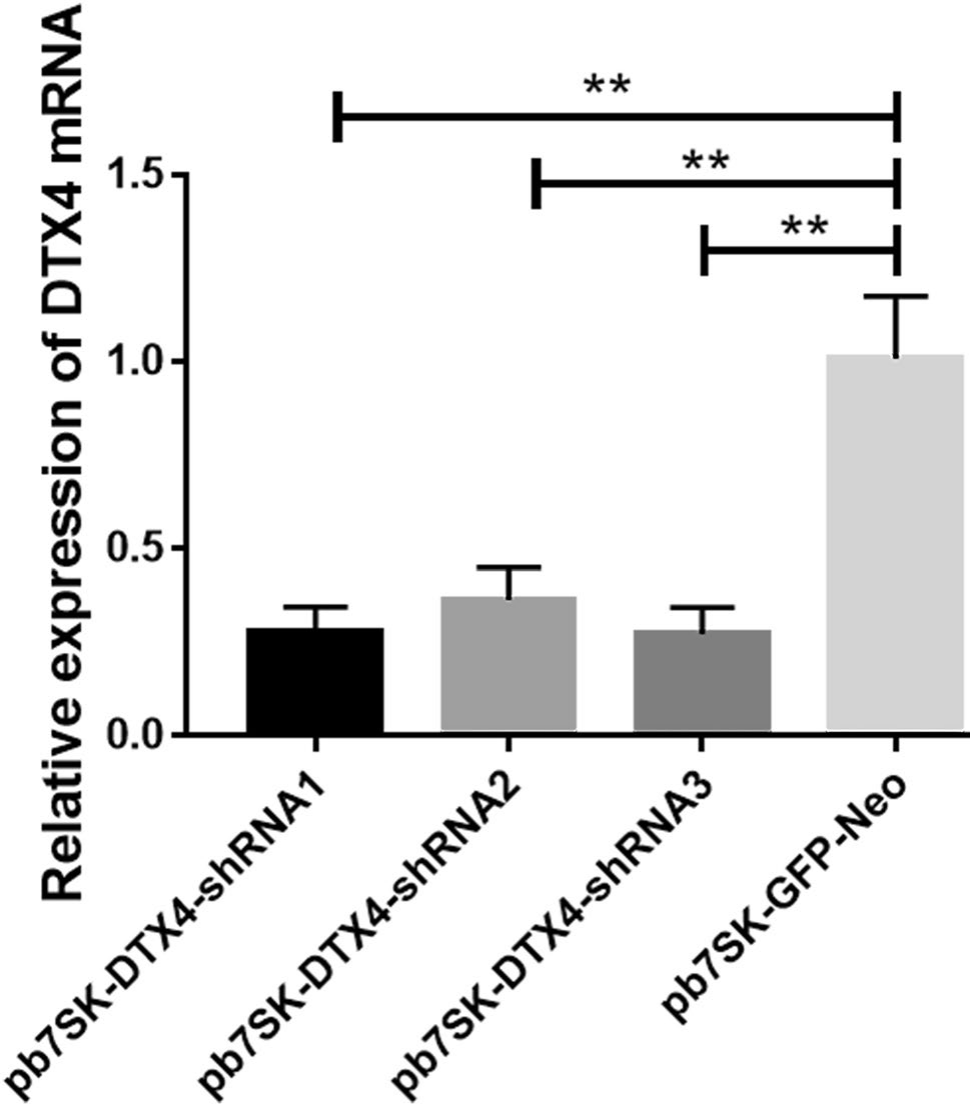
Relative expression levels of *DTX4* mRNA in bMECs transfected with the following vectors: pb7SK-*DTX4*-shRNA1, pb7SK-*DTX4*-shRNA2, pb7SK-*DTX4*-shRNA3, or pb7SK-GFP-Neo (**P < 0.01).

By annealing the *DTX4* interference sequence into the pb7SK vector, pb7SK-*DTX4*-shRNA interference vectors were successfully constructed (Fig. 7). The vectors were transfected into cells, and then the RNA of the cells was extracted 36 h later. The mRNA levels were analysed by reverse transcription and qPCR. qPCR detected that pb7SK-*DTX4*-shRNA3 had the highest interference efficiency among the three designed vectors (Fig. 8).

### Detection of the TGs and CHOL contents in bMECs transfected with the pb7SK-*DTX4*-shRNA vector

**Figure 9:**
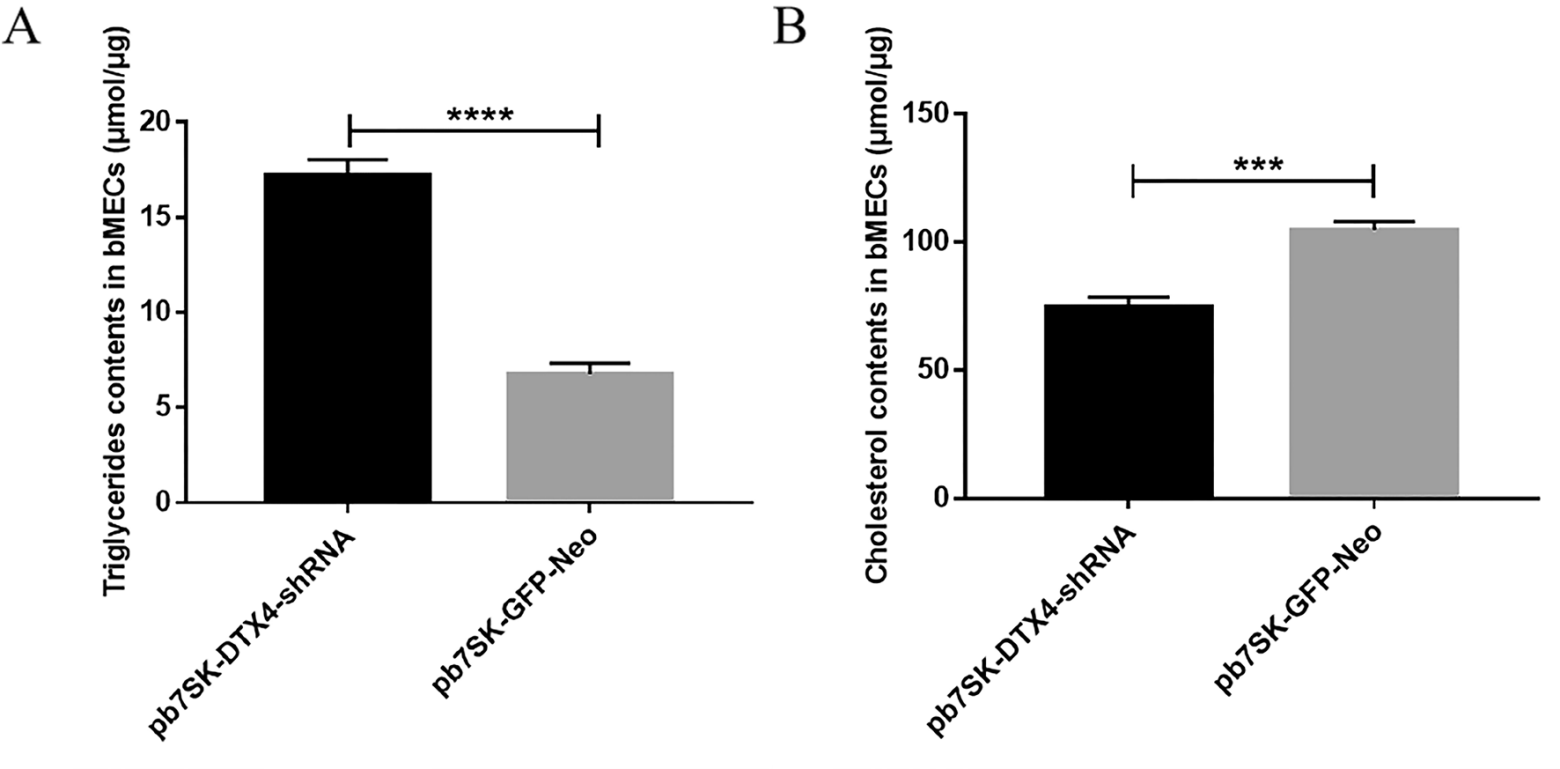
Detection of the TGs and CHOL contents of bMECs after the transfection of pb7SK-*DTX4*-shRNA or pb7SK-GFP-Neo.

To verify the effect of *DTX4* on TGs and CHOL, we transfected bMECs with pb7SK-*DTX4*-shRNA and pb7SK-GFP-Neo. The TGs contents in bMECs of the pb7SK-*DTX4*-shRNA group were significantly increased (**P < 0.01) compared with those of the pb7SK-GFP-Neo group (Fig. 9A). However, the CHOL content in bMECs of the pb7SK-*DTX4*-shRNA group was significantly decreased (**P < 0.01) compared with that in bMECs of the pb7SK-GFP-Neo group (Fig. 9B).

## Discussion

As the human population increases, the demand for milk has also increased. For meat supply, we have many sources, such as chicken, beef, mutton, etc., but milk for human consumption can only be obtained from female mammals, mainly from cows. Milk plays an essential role in the development of bone. Thus, scientists have put significant effort into improving the available cow breeds. Recently, the discovery of functional miRNAs has caused miRNAs to be recognized by an increasing number of scholars as important regulators of lipid metabolism in the body. MiRNAs participate in the regulation of fatty acids and metabolites in many different ways. Many miRNAs are essential to milk fat, lipid metabolism, and mammary gland development. Clusters of MECs are important components of mammary tissue that also have other biological functions. The growth and development of bMECs affect milk production capacity during lactation, and new bMECs formation is critical for maintaining tissue homeostasis^23^. Therefore, bMECs can be used as an important material for studying the mechanism of milk fat synthesis.

In our laboratory, microRNA sequencing was performed on mammary epithelial cells from Chinese Holstein cows with high or low milk fat percentages. MiR-485 was a predicted differentially expressed microRNA that may be involved in the regulation of milk fat metabolism. Bioinformatic analysis showed that the mRNA of the *DTX4* gene had the binding site for miR-485 in the 3′UTR. This was further verified through luciferase reporter assays. BMECs were cultured and transfected with miR-485 mimics, inhibitor, or shNC to determine the effect of modulating miR-485 in these groups. The corresponding assays were performed, including fluorescence imaging, RT-PCR, and Western blotting, to determine the cell number, the relative expression of miR-485 and its target mRNA, *DTX4*, and the protein expression of *DTX4* in bMECs. Cell morphological analysis and fluorescence imaging showed that the transfection was successful.

Furthermore, the expression of miR-485 was higher in the miR-485 mimics group than in the inhibitor and control groups, and its target mRNA, *DTX4*, showed the opposite expression pattern. Additionally, the levels of triglycerides and cholesterol were analysed. The results showed that the levels of TGs and CHOL were higher in the miR-485 mimics group than in the inhibitor and control groups, while the TGs and CHOL contents in the inhibitor group were lower than in the mimics and control groups. This delineates the role of miR-485 in regulating the expression of the *DTX4* gene and ultimately regulating TGs and CHOL production in bMECs. *DTX4* protein expression analysis via Western blot confirmed that the expression of the *DTX4* protein in the miR-485 inhibitor group was significantly higher than that in the miR-485 mimics and miR-485-shNC groups. These results confirm the results of the bioinformatic analysis by revealing the expression pattern of *DTX4* at the mRNA and protein levels and verify that it is a target gene of miR-485.

A previous study showed that miR‐485‐5p suppressed the invasion of cancer cells by targeting *FLOT‐1* in HPV‐infected cervical carcinoma cells^24^. Another study showed that elevated expression of miR‐485‐3p suppressed ki‐67 and cyclin D1 expression in NSCs. The same study also proved that miR‐485‐3p decreased the proliferation and induced the differentiation of NSCs by modulating *TRIP6* expression. This information suggests that miR‐485‐3p has a vital role in the self‐proliferation and differentiation of NSCs. MiR‐485‐3p and *TRIP6* modulation may be promising therapy for treating neurodegenerative and neurogenic diseases^25^. One miRNA can regulate hundreds of genes, one gene may be affected by multiple miRNAs, and miRNAs can upregulate or downregulate gene expression by interacting with target genes. Previous studies have shown that the *DTX4* gene, the adipocyte marker gene *aP2,* and the transcription factor *PPAR-g* all play important roles in the adipogenesis and differentiation of mouse 3T3-L1 preadipocytes and can promote adipogenesis and differentiation processes. This hypothesis was proven by constructing stable *DTX4* knockdown cells with lentivirus and non-targeted control cells. After stable knockdown of *DTX4* was confirmed in the cells, a reduction in lipid droplet synthesis and a reduction in the adipogenic transcription factor PPAR-g and adipocyte marker gene *aP2* were observed, thus proving that *DTX4* is essential for adipogenesis in 3T3-L1 cells^26^. Peroxisome proliferator-activated receptors (PPARs) belong to the ligand-inducible nuclear hormone receptor superfamily and comprise three groups: *PPARα*, *PPARβ/δ PPAR-g*. *PPAR-g* improved adipogenic differentiation, lipid storage, and lipid metabolism^27^. This study provides a possible explanation of why *DTX4* has a role in lipid metabolism through *PPAR-g,* which lays a theoretical foundation for further exploring the biological function of the *DTX4* gene.

Our analysis includes evidence that the NEFA, TGs, and CHOL contents of bMECs are regulated by miR-485 through its effect on the *DTX4* gene. This study also adds new insight into the regulatory pathway of lipid metabolism, providing simple but potentially critical future research directions.

## Conclusion

The results show that miR-485 regulates the synthesis of TGs and CHOL in bMECs by targeting the *DTX4* gene. This study lays the foundation for further research to clarify the molecular mechanism and provides evidence for the in-depth analysis of the function of *DTX4* in milk fat synthesis.

## Materials and methods

### Samples and ethical approval

BMECs were provided by the Animal Science Laboratory of Jilin University, Jilin. According to previous work performed in our laboratory using the tissue nibble culture method, the bMECs in the study were purified and cultured from healthy Chinese Holstein dairy cows. Animal experiments were performed in strict accordance with the Guidelines for the Care and Use of Laboratory Animals of Jilin University (Animal Care and Use Committee permit number: SY201901007)^28,29^. This study only involved bMECs. The experimental design and procedures were performed the following Guangdong Ocean University biological experiment guidelines and regulations. Cow mammary gland tissues were collected under aseptic conditions and stored in 10× penicillin–streptomycin solution PBS buffer; the tissue was soaked in 75% alcohol for 30–60 s and then washed for 3–5 min each in 10×, 5×, and 2× PBS containing penicillin–streptomycin to remove blood and impurities from the tissue. The blood vessels and nerves visible to the naked eye in the tissue block were removed, clean tissue was collected, and the tissue was cut into pieces. bta-miR-485 mimics (AGAGGCTGGCCGTGATGAATTCG), bta-miR-485 inhibitor (AATTCCGAATTCATCACGGCCAGCCTCTCGATCGAATTCATCACGGCCAGCCTCTACCGGTCGAATTCATCACGGCCAGCCTCTTCACCGAATTCATCACGGCCAGCCTCTTTTTTTACCGG), and miRNA-shNC (AAATGTACTGCGCGTGGAGAC) were synthesized by Shanghai Gene Pharma Corporation Technology Company.

### Cell culture, total RNA extraction, and qPCR

The bMECs were cultured in a basal medium consisting of DMEM/F12 (HyClone, Grand Island, NY, USA) supplemented with 10% (v/v) fetal bovine serum and 1% (v/v) penicillin and streptomycin (PAA, Pasching, Austria). The cells were incubated in 5% CO_2_ at 37 °C. The plasmids were transfected into bMECs. Twelve hours before transfection, the bMECs were seeded in six-well cell culture plates, and transfection was performed when the cell confluence reached 80%. The cells were washed twice with PBS, and the PBS was replaced with a fresh medium. The transfection system consisted of 3 μg of plasmids, 7.5 μl of FuGENE HD Transfection Reagent (Promega Biotechnology Company, Beijing, China), and 150 μl of DMEM/F12. The transfected cells were incubated for 15 min at room temperature and then transferred to an incubator. After 36 h, the six-well plates were observed under a fluorescence microscope and photographed to evaluate the transfection efficiency.

RNA extraction was performed on the cells using TRIzol (15596-026, Invitrogen, USA). A spectrophotometer (Thermo, Nanodrop-2000) was used to determine the concentration and purity of total RNA. The concentration of the mimics was 293.1 ng/μl, and the OD was 1.80. The concentration of the inhibitor was 342.7 ng/μl, and the OD was 1.83. The RNA concentration in shNC was 189.0 ng/μl, and the OD was 1.71. After that, reverse transcription of the extracted RNA was performed using a Reverse Transcription Kit (RR047A, Takara Bio, Dalian, China), and then qRT-PCR SYBR Fluorescent Dye (RR420A, Takara Bio, Beijing, China) was used to examine the relative expression levels in the cells. All experiments were repeated three times.

### Detection of TGs, CHOL, and NEFA

The miR-485 plasmids were transfected into bMECs, and then the TGs, CHOL, and NEFA contents were extracted 36 h later. We used a tissue TG Assay Kit (APPLYGEN, E1013, Beijing, China), Tissue CHOL Assay Kit (APPLYGEN, E1015, Beijing, China), NEFA Detection Kit (Nanjing Jiancheng Bioengineering Institute, A042-2-1, Nanjing, China), and BCA Protein Quantification Kit (Vazyme, E112-02, Nanjing, China) to quantify TGs, CHOL, and NEFA in bMECs. Finally, we used a microplate reader (SYNERGY HTX multi-mode reader, Bio Tek, S1LFTA) to measure the absorbance of the samples.

### Bioinformatics prediction of miR-485 target genes

The miRbase (www.mirbase.org/) software was used to select the mature sequence of miR-485 for the prediction of target genes. The target genes and binding sites of miR-485 potentially related to milk fat synthesis were predicted by TargetScan (http://www.targetscan.org/vert_72/), and qPCR primer sequences were designed and synthesized according to miRNA primer design principles (synthesized by GENEWIZ, Suzhou, China) (Table 1).

**Table 1:** Real-time PCR primer sequences.

Symbol	Primer	Primer Sequence (5′–3′)
bta-miR-485	RT-Primer	GTCGTATCCAGTGCAGGGTCCGAGGTATTCGCACTGGATACGACCGAATTCA
	F-Primer	TGCGGAGAGGCTGGCCGTGATG
	R-Primer	CAGTGCAGGGTCCGAGGT
U6	RT-Primer	AACGCTTCACGAATTTGCGT
	F-Primer	CTCGCTTCGGCAGCACA
	R-Primer	AACGCTTCACGAATTTGCGT
DTX4	F-Primer	AGAAGAAGTGCTGAAGAAGT
	R-Primer	ATCCTTGTTGCCATTGTTG
β-actin	F-Primer	AGAGCAAGAGAGGCATCC
	R-Primer	TCGTTGTAGAAGGTGTGGT

### Luciferase reporter assay

The miR-485 binding site sequence of *DTX4* was amplified by PCR for *NotI* (0551712, Biolabs, America) and *XhoI* (K2704AA, TAKARA, Beijing, China) restriction fragments. We cloned the sequences into the pmiR-RB-reporter vector to construct the pmiR-RB-report-*DTX4* wt/mut/si vectors. All the sequence details of the DNA oligos are listed in Table 2. Plasmids were constructed by annealing, restriction digestion, ligation, transformation, and other methods (Endo Free Maxi Plasmid Kit, Tiangen Biotech Company, DP117, Beijing, China). The pmiR-BR-REPORT-*DTX4*-wt/mut/si vectors were cotransfected into the bMECs of the miR-485 mimics group. The firefly (hluc+) and Renilla (hRluc) luciferase activities were detected by the Dual-Glo Luciferase Assay System (Cat. #E1960, Promega, USA) according to the manufacturer’s protocols; hluc+ luciferase was used as the reference to correct for variations in transfection efficiency, and the relative luciferase activity was calculated with the following equation: relative luciferase activity = hRluc/hluc+. Three replicates of the experiments were carried out by transfecting equal numbers of cells using the same vectors in various wells. The fluorescence values were detected by a SpectraMax M5 microplate reader to determine whether the 3′UTR of the *DTX4* gene contains binding sites for miR-485.

**Table 2:** Primer sequences.

Symbol	Primer	Primer sequence (5′–3′)
*DTX4*-WT	F-Primer	GGCCCAGGAGTGATGATCTTGGTTGTTCTCAGCCTCATCAGTTTTCCCCAAAATTACTA
	R-Primer	TCGATAGTAATTTTGGGGAAAACTGATGAGGCTGAGAACAACCAAGATCATCACTCCTG
*DTX4*-mut	F-Primer	GGCCCAGGAGTGATGATCTTGGTTGTTCTACTCCGACTCAGTTTTCCCCAAAATTACTA
	R-Primer	TCGATAGTAATTTTGGGGAAAACTGAGTCGGAGTAGAACAACCAAGATCATCACTCCTG

### Western blot analysis

Bta-miR-485 mimics, bta-miR-485 inhibitor, and shNC were transfected into bMECs for 36 h, and then the total protein was extracted. The protein was extracted by RIPA lysis buffer (Meilun Biotechnology Company, P.N.: MA0171, Dalian, China). The cell lysate was centrifuged at 12,000 rpm at 4 °C for 15 min, and the supernatant was collected. Protein concentration was determined with the bicinchoninic acid (BCA) protein quantitation assay (Vazyme Biotech Company, E112-01/02, Nanjing, China). We used a PAGE Gel Rapid Preparation Kit (EpiZyme, Biotechnology Company, LOT: 01381306P, Shanghai, China) to prepare separation gels and concentrated gels. We incubated the membrane with anti-DTX4 antibody (bs-14439R-HRP, Beijing Biosynthetic Biotechnology Company, China) at a dilution of 1:100, anti-β-actin polyclonal antibody (bs-0061R, Beijing Biosynthetic Biotechnology Company, China) and Goat Anti-Rabbit IgG Antibody (H + L), HRP Conjugated (bs-0295G-HRP, Beijing Biosynthetic Biotechnology Company, China). The Tanon-4600 fluorescence/chemiluminescence analysis system was used to capture the signal intensity to obtain the gray value ratio.

### Construction of *DTX4* gene interference vectors

Three pairs of shRNA sequences were designed through the Thermo website (https://rnaidesigner.thermofisher.com/rnaiexpress/), and the shRNAs were synthesized by Shenggong Biotechnology Company. The shRNA sequences are shown in Table 3. Annealed siRNA oligonucleotides were cloned into a modified pb7SK-GFP-Neo vector. Then, we selected the interference vector with the best interference efficiency.

**Table 3:** Primer sequences.

Symbol	Primer	Primer sequence (5′–3′)
*DTX4*-7sk-sh1	F-Primer	AGAGGCCACGTCTACCACGTCTATTTCAAGAGAATAGACGTGGTAGACGTGGCTTTTTTG
	R-Primer	GATCCAAAAAAGCCACGTCTACCACGTCTATTCTCTTGAAATAGACGTGGTAGACGTGGC
*DTX4*-7sk-sh2	F-Primer	AGAGGCAGAACCATCCGGATCATCTTCAAGAGAGATGATCCGGATGGTTCTGCTTTTTTG
	R-Primer	GATCCAAAAAAGCAGAACCATCCGGATCATCTCTCTTGAAGATGATCCGGATGGTTCTGC
*DTX4*-7sk-sh3	F-Primer	AGAGGGAATGAAGTCCATCACAAGATCAAGAGTCTTGTGATGGACTTCATTCCTTTTTTG
	R-Primer	GATCCAAAAAAGGAATGAAGTCCATCACAAGACTCTTGATCTTGTGATGGACTTCATTCC

### Statistics and analysis

The data were analysed by GraphPad Prism 6 software. The mean and standard error (SE) of the mean were calculated from triplicate wells. *P* < 0.05 was considered statistically significant. For relative quantitation, the 2^−ΔΔCt^ method was used.

## Supplementary Information

Supplementary Information.
