# Supplementary material for: MiR-485 targets the DTX4 gene to regulate milk fat synthesis in bovine mammary epithelial cells
Source: Sci Rep. 2021 Apr 7;11:7623. doi: 10.1038/s41598-021-87139-5 (PMC8027660; doi:10.1038/s41598-021-87139-5)

- According to reviewers suggestion, we provide original western blot pictures to prove the correctness of the results.

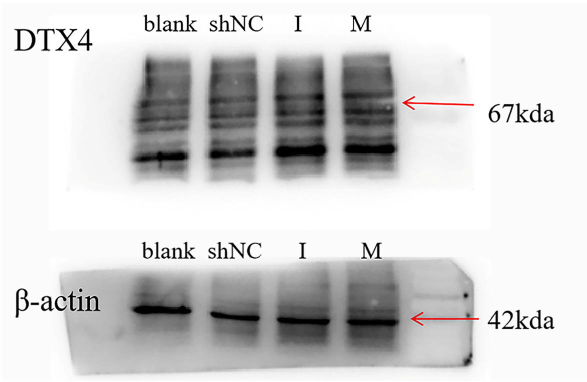

- According to reviewers suggestion, we provide to we checked other lipogenic gene include MVD,HMGCS1,FDFT1 at mRNA level. The result of all these three gene was significant. We provided the result of these three gene in supplement picture.

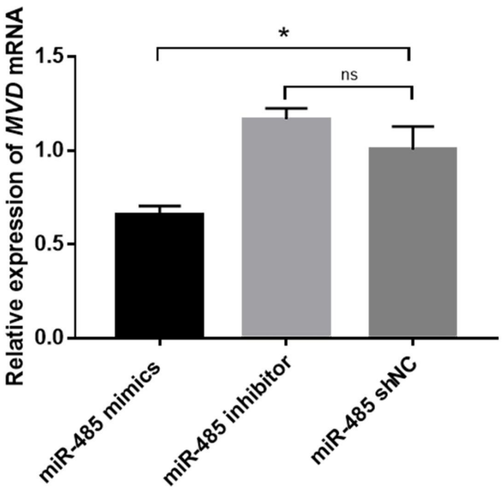

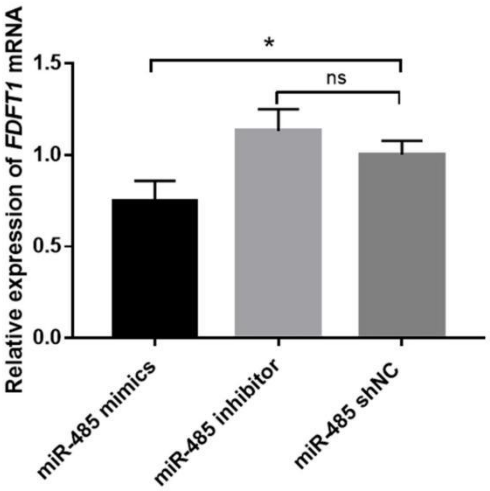

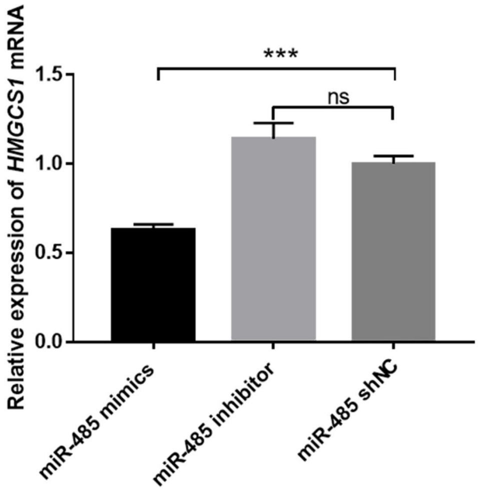

Supplement: Supplementary file 1 — Supplementary Information. [file 41598_2021_87139_MOESM1_ESM.pdf]
